# Supplementary material for: GATI-RS model using Bi-LSTM and multi-head attention mechanism to enhance online shopping experience for the elderly with accurate click-through rate prediction
Source: PeerJ Comput Sci. 2025 Feb 20;11:e2707. doi: 10.7717/peerj-cs.2707 (PMC11888906; doi:10.7717/peerj-cs.2707)
Supplement: Supplemental Information 2 [file peerj-cs-11-2707-s002.pdf]

Reproducibility

Computing Infrastructure: The experiments were conducted on a system running Windows OS, with hardware configurations including an Intel Core i7-9700K CPU, 16GB RAM, and an NVIDIA GeForce RTX 2070 GPU. We used Python 3.8 and TensorFlow 2.x for development and experiments.

Model Description: This study employs a recommendation system model combining Bi-LSTM and a multi-head self-attention mechanism. The model is designed to predict click-through rates (CTR) by analyzing user characteristics, product features, and their interactions.

Evaluation Method: Accuracy (ACC) and Loss (LOSS) were used as the primary evaluation metrics. The best model was selected based on its performance on the validation set, utilizing an early stopping strategy (5 epochs without improvement).

Assessment Metrics (Justification): Accuracy and loss functions were chosen as evaluation metrics because they directly reflect the performance of the classification model and the convergence of the training process.

Limitations/Validity: We discussed the generalization ability of the model on different datasets and its potential limitations. The effectiveness of the model was validated through extensive experiments.

Code and Data Availability: The code used in this study will be provided as supplementary files. A direct download link for the dataset will also be included to ensure the reproducibility of the results.
